# Supplementary material for: Empirical methods for controlling false positives and estimating confidence in ChIP-Seq peaks
Source: BMC Bioinformatics. 2008 Dec 5;9:523. doi: 10.1186/1471-2105-9-523 (PMC2628906; doi:10.1186/1471-2105-9-523)
Supplement: Additional File 1 — A variety of html documents from the USeq web site detailing the available applications, their best usage, output file type descriptions, command line menus, etc. [file 1471-2105-9-523-S1.zip › USeqUserGuides/JavaDoc.zip!/JavaDoc/trans/graphics/class-use/GenomicRegion.html]

Uses of Class trans.graphics.GenomicRegion


|  |  |  |
| --- | --- | --- |
| **Overview** \| **Package** \| **Class** \| **Use** \| **Tree** \| **Deprecated** \| **Index** \| **Help** | |  |
| PREV   NEXT | **FRAMES**    **NO FRAMES**     **All Classes** |  |


---


## **Uses of Class trans.graphics.GenomicRegion**

| Packages that use GenomicRegion | |
| --- | --- |
| **trans.graphics** | For graphically representing '.cel' files and intervals. |

Uses of GenomicRegion in trans.graphics

| Methods in trans.graphics that return GenomicRegion | |
| --- | --- |
| `GenomicRegion` | `RankedSetDrawPanel.findRegion(GenomicRegion[] regions, java.awt.geom.Point2D point)`             Scans an array of GenomicRegions Rectangles to see if the Point is present, ie they clicked it. |
| `GenomicRegion[]` | `RankedSetAnalysis.getRegionsOne()` |
| `GenomicRegion[]` | `RankedSetAnalysis.getRegionsTwo()` |
| `static GenomicRegion[]` | `RankedSetAnalysis.parseGenomicRegionsFile(java.io.File f)`             Parses a regions file. |

| Methods in trans.graphics with parameters of type GenomicRegion | |
| --- | --- |
| `static boolean` | `RankedSetAnalysis.containsMatchedRankIntersect(GenomicRegion r, int testRank)`             Checks to see if among the hits to a particular GenomicRegion they have an equal or smaller rank. |
| `static int` | `GenomicRegion.findBiggestGenomicRegion(GenomicRegion[] gr)`             Returns the length of the biggest GenomicRegion |
| `GenomicRegion` | `RankedSetDrawPanel.findRegion(GenomicRegion[] regions, java.awt.geom.Point2D point)`             Scans an array of GenomicRegions Rectangles to see if the Point is present, ie they clicked it. |
| `java.lang.String` | `RankedSetDrawPanel.formattedInfo(GenomicRegion g)` |
| `double` | `RankedSetAnalysis.fractionTotalIntersection(GenomicRegion[] r)`             Returns the total fraction of intersection. |
| `boolean` | `GenomicRegion.overlap(GenomicRegion other, int maxGap)`             checks to see if regions overlap by the minimum maxGap, can set maxGap negative to require an overlap. |
| `void` | `RankedSetAnalysis.printRankedIntersectionAll(GenomicRegion[] r)`             Rank analysis where each regions is check against the entire other list. |
| `static int[]` | `RankedSetAnalysis.rankedIntersection(GenomicRegion[] r)`             Calculates the number of hits in the ranked matched list. |

---


|  |  |  |
| --- | --- | --- |
| **Overview** \| **Package** \| **Class** \| **Use** \| **Tree** \| **Deprecated** \| **Index** \| **Help** | |  |
| PREV   NEXT | **FRAMES**    **NO FRAMES**     **All Classes** |  |


---
